# Differentially conserved amino acid positions may reflect differences in SARS-CoV-2 and SARS-CoV behaviour

**DOI:** 10.1093/bioinformatics/btab094

**Published:** 2021-02-09

**Authors:** Denisa Bojkova, Jake E McGreig, Katie-May McLaughlin, Stuart G Masterson, Magdalena Antczak, Marek Widera, Verena Krähling, Sandra Ciesek, Mark N Wass, Martin Michaelis, Jindrich Cinatl

**Affiliations:** Institute for Medical Virology, University Hospital, Goethe University Frankfurt am Main, Frankfurt am Main 60596, Germany; School of Biosciences, University of Kent, Canterbury CT2 7NJ, UK; Institute of Virology, Biomedical Research Center (BMFZ), Philipps University Marburg, Marburg 35037, Germany; School of Biosciences, University of Kent, Canterbury CT2 7NJ, UK; School of Biosciences, University of Kent, Canterbury CT2 7NJ, UK; Institute of Virology, Biomedical Research Center (BMFZ), Philipps University Marburg, Marburg 35037, Germany; Institute of Virology, Biomedical Research Center (BMFZ), Philipps University Marburg, Marburg 35037, Germany; Institute for Medical Virology, University Hospital, Goethe University Frankfurt am Main, Frankfurt am Main 60596, Germany; German Center for Infection Research, DZIF, Braunschweig 60596, Germany; School of Biosciences, University of Kent, Canterbury CT2 7NJ, UK; School of Biosciences, University of Kent, Canterbury CT2 7NJ, UK; Institute for Medical Virology, University Hospital, Goethe University Frankfurt am Main, Frankfurt am Main 60596, Germany

## Abstract

**Motivation:**

SARS-CoV-2 is a novel coronavirus currently causing a pandemic. Here, we performed a combined in-silico and cell culture comparison of SARS-CoV-2 and the closely related SARS-CoV.

**Results:**

Many amino acid positions are differentially conserved between SARS-CoV-2 and SARS-CoV, which reflects the discrepancies in virus behaviour, i.e. more effective human-to-human transmission of SARS-CoV-2 and higher mortality associated with SARS-CoV. Variations in the S protein (mediates virus entry) were associated with differences in its interaction with ACE2 (cellular S receptor) and sensitivity to TMPRSS2 (enables virus entry via S cleavage) inhibition. Anti-ACE2 antibodies more strongly inhibited SARS-CoV than SARS-CoV-2 infection, probably due to a stronger SARS-CoV-2 S-ACE2 affinity relative to SARS-CoV S. Moreover, SARS-CoV-2 and SARS-CoV displayed differences in cell tropism. Cellular ACE2 and TMPRSS2 levels did not indicate susceptibility to SARS-CoV-2. In conclusion, we identified genomic variation between SARS-CoV-2 and SARS-CoV that may reflect the differences in their clinical and biological behaviour.

**Supplementary information:**

Supplementary data are available at *Bioinformatics* online.

## 1 Introduction

In December 2019, severe acute respiratory syndrome coronavirus 2 (SARS-CoV-2), a novel betacoronavirus, was identified that causes a respiratory disease and pneumonia called coronavirus disease 19 (COVID-19) (Coronaviridae Study Group of the International Committee on Taxonomy of Viruses, 2020; Zhu et al., 2020). As of 22nd of December 2020, 77 801 721 confirmed COVID-19 cases and 1 713 109 COVID-19 deaths have been reported (Dong *et al.*, 2020). Since 2002, SARS-CoV-2 is the third betacoronavirus, after severe acute respiratory syndrome coronavirus (SARS-CoV) and Middle East respiratory syndrome coronavirus (MERS-CoV), that has caused a substantial outbreak associated with significant mortality (Wu *et al.*, 2020).

SARS-CoV-2 is closely related to SARS-CoV (Coronaviridae Study Group of the International Committee on Taxonomy of Viruses, 2020; Wu *et al.*, 2020). Entry of both viruses is mediated via interaction of the viral Spike (S) protein with the cellular receptor ACE2, and both viruses depend on S activation by cellular proteases, in particular by TMPRSS2 (Cui *et al.*, 2019; Hoffmann *et al.*, 2020a; Walls *et al.*, 2020; Wan et al., 2020; Wrappet al., 2020; Wu *et al.*, 2020; Yan et al., 2020). Despite these similarities, the diseases caused by SARS-CoV-2 (COVID-19) and SARS-CoV (SARS) differ. According to WHO, the SARS-CoV outbreak resulted in 8098 confirmed and suspected cases and 774 deaths, equalling a mortality rate of 9.6% (www.who.int). Estimated mortality rates for SARS-CoV-2 are below 1% (Borges do Nascimento, 2020). SARS-CoV was only spread by symptomatic patients with severe disease (Cheng *et al.*, 2013). In contrast, SARS-CoV-2 has been reported to be transmitted by individuals who are asymptomatic during the incubation period or who do not develop symptoms at all (Rivett*et al.*, 2020).

We have developed an approach to identify sequence-associated phenotypic differences between related viruses based on the identification of differentially conserved amino acid sequence positions (DCPs) and in silicomodelling of protein structures (Martell *et al.*, 2019; Pappalardo*et al.*, 2016). Conserved amino acid positions are likely to be of functional relevance, and differential conservation may indicate functional differences and they have been widely used for the analysis of protein families (Rausell*et al.*, 2010, Das *et al*., 2015). Here, we used this method to identify differentially conserved positions that may explain phenotypic differences between SARS-CoV-2 and SARS-CoV. These data were combined with data derived from virus-infected cells.

## 2 Materials and methods

### 2.1 Structural analysis

Sequences for each of the SARS-CoV-2 proteins were obtained from the GISAID resource. The protein sequences were then filtered for sequences from human hosts with high coverage, and sequences with spans of X’s were removed. The number of sequences retained after filtering for each protein is shown in Supplementary Table S4. Fifty-three SARS-CoV genome sequences derived from human hosts were downloaded from VIPR (Pickett *et al.*, 2012a,b). Open Reading Frames (ORFs) were extracted using EMBOSS getorf (Rice *et al.*, 2000) and matched to known proteins using BLAST. Fragments and mismatches were discarded. To match the ORF1ab non-structural proteins, a BLAST database of the sequences from the SARS non-structural proteins was generated and the SARS-CoV-2 ORF1ab searched against it. The sequences for each protein were then aligned using ClustalO (Sievers*et al.*, 2011) with default settings.

Conserved positions were identified by calculating the Jensen-Shannon divergence score (Capra & Singh, 2007) for each position in the multiple sequence alignment in virus. Differing alignment positions with conservation score >0.8 for both species were considered as differentially conserved positions (DCPs).

SARS-CoV-2 and SARS-CoV protein structures were downloaded from the Protein Databank (PDB; Supplementary Table S1) (Armstrong *et al.*, 2020). Where structures were not available, they were modelled using Phyre2 (Kelley *et al.*, 2015; Supplementary Table S2). Where Phyre2 did not generate a confident model, structural models from AlphaFold were used (Senior *et al.*, 2020). Ligand binding sites were modelled using 3DLigandSite (Wasset al., 2010). DCPs were mapped onto protein structures using PyMOL. Exposed (solvent-accessible) and buried (solvent-inaccessible) residues were identified using Python module *findSurfaceResidues* with default parameters. Amino acid changes at DCPs were manually analysed for their potential impact on protein structure and function based on the presence or absence of hydrogen bonding, changes in hydrogen bonding capacity and changes in charge in SARS-CoV compared with SARS-CoV-2 proteins. Where models were unavailable, mutagenesis was performed within PyMOL to assess the potential impact of the amino acid changes. The structural analysis grouped DCPs into six different categories based on the effect that they were proposed to have. These include ‘unlikely’, ‘possible’ and ‘likely’. The possible and likely categories were split into three and two subgroups respectively depending on the type of effect (Supplementary Table S3).

### 2.2 Cell culture

The Caco2 cell line was obtained from DSMZ (Braunschweig, Germany). The cells were grown at 37°C in minimal essential medium (MEM) supplemented with 10% foetal bovine serum (FBS), 100 IU/ml penicillin, and 100 μg/mL of streptomycin. 293 cells (PD-02-01; MicrobixBisosystems Inc.) and 293/ACE2 cells (Kamitani *et al.*, 2006) (kindly provided by Shinji Makino, UTMB, Galveston, Texas) were cultured in Dulbecco’s modified Eagle medium (DMEM) supplemented with 10% FBS, 50 IU/mL penicillin and 50 µg/mL streptomycin. Selection of 293/ACE2 cells constitutively expressing human angiotensin-converting enzyme 2 (ACE2) was performed by addition of 12 µg/mL blasticidin. All culture reagents were purchased from Sigma (Munich, Germany). Cells were regularly authenticated by short tandem repeat (STR) analysis and tested for mycoplasma contamination.

### 2.3 Virus infection

The isolate SARS-CoV-2/1/Human/2020/Frankfurt (Hoehl*et al.*, 2020) was cultivated in Caco2 cells as previously described for SARS-CoV strain FFM-1 (Cinatl*et al.*, 2004). Virus titres were determined as TCID50/ml in confluent cells in 96-well microtitre plates (Cinatl*et al.*, 2003; 2005).

### 2.4 Western blot

Western blotting was performed as previously described (Schneider *et al.* 2017). Briefly, cells were lysed using Triton-X-100 sample buffer, and proteins were separated by SDS-PAGE. Proteins were blotted on a nitrocellulose membrane (Thermo Scientific). Detection occurred by using specific antibodies against β-actin (1:2500 dilution, Sigma-Aldrich, Munich, Germany), ACE2 and TMPRSS2 (both 1:1000 dilution, abcam, Cambridge, UK) followed by incubation with IRDye-labeled secondary antibodies (LI-COR Biotechnology, IRDye^®^800CW Goat anti-Rabbit, 926-32211, 1:40 000) according to the manufacturer’s instructions. Protein bands were visualized by laser-induced fluorescence using infrared scanner for protein quantification (Odyssey, Li-Cor Biosciences, Lincoln, NE, USA).

### 2.5 Receptor blocking experiments

SARS-CoV/SARS-CoV-2 receptor blocking experiments were adapted from Cinatl*et al* (2004). Caco2 cells were pre-treated for 30 min at 37°C with goat antibodies directed against the human ACE2 or DDP4 ectodomain (R&D Systems, Wiesbaden-Nordenstadt, Germany). Then, cells were washed three times with PBS and infected with SARS-CoV-2 at MOI 0.01. Cytopathogenic effects were monitored 48 h post-infection. Cytopathogenic effect (CPE) was assessed visually by light microscopy by two independent laboratory technicians 48 h after infection (Cinatl*et al.*, 2003).

### 2.6 Antiviral assay

Confluent cell cultures were infected with SARS-CoV-2 or SARS-CoV in 96-well plates at MOI 0.01 in the absence or presence of drug. Cytopathogenic effect (CPE) was assessed visually by light microscopy by two independent investigators 48 h post-infection (Cinatl*et al.*, 2003).

### 2.7 Viability assay

Cell viability was determined by 3-(4,5-dimethylthiazol-2-yl)-2,5-diphenyltetrazolium bromide (MTT) assay modified after Mosmann (Mosmann, 1983), as previously described (Onafuye*et al.*, 2019).

### 2.8 Qpcr

SARS-CoV-2 and SARS-CoV RNA was isolated from cell culture supernatants using AVL buffer and the QIAamp Viral RNA Kit (Qiagen) according to the manufacturer’s instructions. RNA was subjected to OneStepqRT-PCR analysis using the SYBR green based Luna Universal One-Step RT-qPCR Kit (New England Biolabs) and a CFX96 Real-Time System, C1000 Touch Thermal Cycler. Primers were adapted from the WHO protocol (Corman*et al.*, 2020) targeting the open reading frame for RNA-dependent RNA polymerase (RdRp) of both SARS-CoV-2 and SARS-CoV: RdRP_SARSr-F2 (GTGARATGGTCATGTGTGGCGG) and RdRP_SARSr-R1 (CARATGTTAAASACACTATTAGCATA) using 0.4 μM per reaction. RNA copies/ml were determined by standard curves which were using plasmid DNA (pEX-A128-RdRP) harbouring the corresponding amplicon regions for SARS-CoV-2 RdRP target sequence (GenBank Accession number NC_045512). For each condition, three biological replicates were used. Mean and standard deviation were calculated for each group.

## 3 Results

### 3.1 Determination of differentially conserved positions (DCPs)

Coronavirus genomes harbour single-stranded positive sense RNA (+ssRNA) of about 30 kilobases in length, which contain six or more open reading frames (ORFs) (Cui *et al.*, 2019; Wu *et al.*, 2020). The SARS-CoV-2 genome has a size of approximately 29.8 kilobases and was annotated to encode 14 ORFs and 27 proteins (Wu *et al.*, 2020). Two ORFs at the 5’-terminus (ORF1a, ORF1ab) encode the polyproteins pp1a and pp1b, which comprise 15 non-structural proteins (nsps), the nsps 1 to 10 and 12–16 (Wu *et al.*, 2020). Additionally, SARS-CoV-2 encodes four structural proteins (S, E, M, N) and eight accessory proteins (3a, 3b, p6, 7a, 7b, 8b, 9b, orf14) (Wu *et al.*, 2020). This set-up resembles that of SARS-CoV. The 8a protein in SARS-CoV is absent in SARS-CoV-2. 8 b is longer in SARS-CoV-2 (121 amino acids) than in SARS-CoV (84 amino acids), while 3 b is shorter in SARS-CoV-2 (22 amino acids) than in SARS-CoV (154 amino acids) (Wu *et al.*, 2020).

To identify genomic differences between SARS-CoV-2 and SARS-CoV that may affect the structure and function of the encoded virus proteins, we identified differentially conserved amino acid positions (DCPs) (Rausell*et al.*, 2010) and determined their potential impact by in silicomodelling (Martell *et al.*, 2019; Pappalardo*et al.*, 2016).

In the reference sequences of the 22 SARS-CoV-2 virus proteins that could be compared with SARS-CoV, 1393 positions encoded different amino acids. 891 (64%, 9% of all SARS-CoV-2 genome residues) of these positions were DCPs (Supplementary Table S2). Most of the amino acid substitutions at DCPs appear to be fairly conservative as demonstrated by the average BLOSUM substitution score of 0.32 (median 0; Supplementary Fig. S1) and with 69% of them having a score of 0 or greater (the higher the score the more frequently such amino acid substitutions are observed naturally in evolution). 46% of DCPs represent conservative changes where amino acid properties are retained (e.g. change between two hydrophobic amino acids), 18% represented polar—hydrophobic substitutions, and <10% were changes between charged amino acids (Supplementary Table S3).

Six of the SARS-CoV-2 proteins have a higher proportion of DCPs, S, 3a, p6, nsp2, nsp3 (papain-like protease), and nsp4 with 14.82%, 11.68%, 9.52%, 21.38%, 17.9% and 10.8% of their residues being DCPs, respectively (Supplementary Table S4). Very few DCPs were observed in the envelope (E) protein and most of remaining non-structural proteins encoded by ORF1ab. For example, no residues in the helicase and <4% of residues in the RNA-directed RNA polymerase, 2’-O-Methyltransferase, nsp8 and nsp9 are DCPs (Supplementary Table S1).

We were able to map 572 DCPs onto protein structures (Supplementary Fig. S2, Supplementary Table S5 and S6). Nearly all of the mapped DCPs occur on the protein surface (86%), with only 34 DCPs buried within the protein, primarily in S and the papain-like protease (nsp3) (Supplementary Table S3). We propose that 49 DCPs are likely to result in structural/functional differences between SARS-CoV and SARS-CoV-2 proteins. A further 259 could result in some change. The remaining 264 DCPs seem unlikely to have a substantial functional impact (Supplementary Table S3).

### 3.2 Differentially conserved positions (DCPs) in interferon antagonists

At least 10 SARS-CoV proteins have roles in interferon antagonism (Totura and Baric, 2012). Two of these proteins, p6 and the papain-like protease (nsp3), contain many DCPs, two have very few DCPs (nsp7 and nsp16), five have intermediate numbers of DCPs (nsp14, nsp1, nsp15, N and M), while p3b is not encoded by SARS-CoV-2. Initial studies have identified a difference in the interferon inhibition between SARS-CoV and SARS-CoV-2 (Lokugamage*et al.*, 2020). Thus, it is possible that especially the DCPs in p6 and the papain-like protease may have an effect on interferon inhibition.

### 3.3 Differences in cell tropism between SARS-CoV-2 and SARS

**Fig 1:**
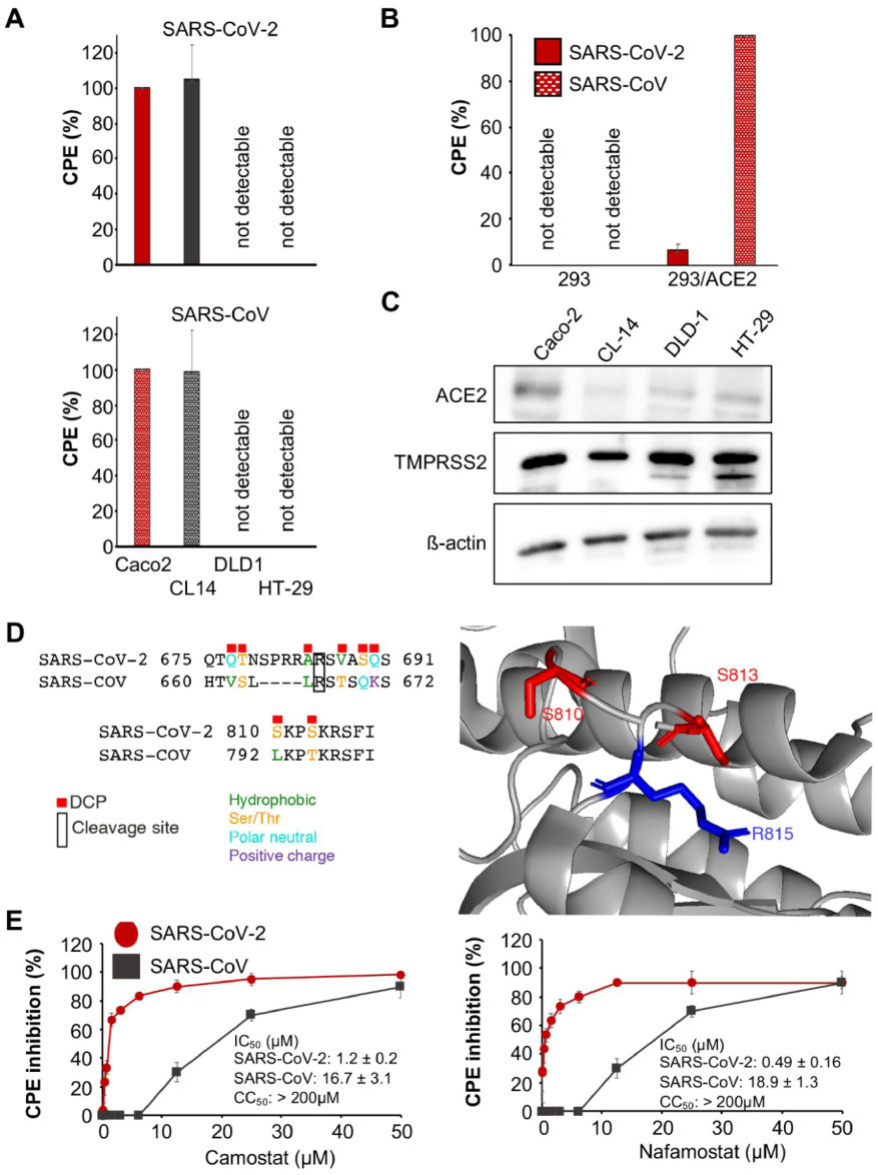
SARS-CoV-2 and SARS-CoV replication in cell culture. (**A**) Cytopathogenic effect (CPE) formation 48 h post-infection in MOI 0.01-infected Caco2, CL14, DLD-1 and HT29 cells. Representative images showing immunostaining for double-stranded RNA (indicates virus replication) and quantification of virus genomes by qPCR are presented in Supplementary Figure S3. (**B**) CPE formation in SARS-CoV and SARS-CoV-2 (MOI 0.01)-infected ACE2-negative 293 cells and 293 cells stably expressing ACE2 cells (293/ACE2) 48 h post-infection. Immunostaining for double-stranded RNA and quantification of virus genomes by qPCR is shown in Supplementary Figure S4. (**C**) Western blots indicating cellular ACE2 and TMPRSS2 protein levels in uninfected cells. Uncropped blots are provided in Supplementary Figure S5. (**D**) A sequence view of the DCPs in the vicinity of the S two cleavage sites and an image of the R815 cleavage site and closely located DCPs. S is cleaved and activated by TMPRSS2. (**E**) Concentration-dependent effects of the TMPRSS2 inhibitors camostat and nafamostat on SARS-CoV-2- and SARS-CoV-induced cytopathogenic effect (CPE) formation determined 48 h post-infection in Caco2 infected at an MOI of 0.01 using a phase contrast microscope. Similar effects were observed in CL14 cells (Supplementary Fig.S6). Values are presented as means ± S.D. (n = 3)

Next, we elucidated whether the substantial number of DCPs results in different phenotypes in cell culture, using the cell lines Caco2, CL14 (susceptible to SARS-CoV infection), HT-29 and DLD-1 (non-susceptible) (Cinatl*et al.*, 2004). Analogously to SARS-CoV infection, SARS-CoV-2 replication was detected in Caco2 and CL14 cells, but not in HT-29 or DLD-1 cells, as shown by cytopathogenic effects (CPE) (Fig. 1A),staining for double-stranded RNA (Supplementary Fig. S3A) and viral genomic RNA levels (Supplementary Fig. S3B).

However, ACE2-expressing 293 cells differed in their susceptibility to SARS-CoV-2 and SARS-CoV (Fig. 1B, Supplementary Fig. S4). ACE2 has been identified as a cellular receptor for both SARS-CoV-2 and SARS-CoV(Cui *et al.*, 2019; Hoffmann *et al.*, 2020a; Walls *et al.*, 2020; Wan *et al.*, 2020; Wrapp*et al.*, 2020; Wu *et al.*, 2020; Yan *et al.*, 2020). Unmodified 293 cells are not susceptible to SARS-CoV infection due to a lack of ACE2 expression. However, 293 cells that stably express ACE2 (293/ACE2) support SARS-CoV infection (Kamitani*et al.*, 2006). As expected, infection of 293 cells with SARS-CoV or SARS-CoV-2 did not result in detectable cytopathogenic effect (CPE) (Fig. 1B), but a SARS-CoV-induced CPE was detected in 293/ACE2 cells (Fig. 1B). In contrast, 293/ACE2 cells displayed limited permissiveness to SARS-CoV-2 infection (Fig. 1B). Staining for double-stranded RNA (Supplementary Fig.S4A) and detection of viral genomic RNA copies (Supplementary Fig.S4B) confirmed these findings. Hence, the ACE2 status does not reliably predict cell sensitivity to SARS-CoV-2. Indeed, CL-14 was characterized by lower ACE2 levels than DLD-1 and HT29 (Fig. 1C).

SARS-CoV-2 and SARS-CoV cell entry depends on S cleavage by transmembrane serine protease 2 (TMPRSS2) (Hoffmann *et al.*, 2020a,b; Zhou et al., 2015). However, the non-SARS-CoV-2 susceptible and susceptible cell lines displayed similar TMPRSS2 levels (Fig. 1C). Thus, cellular TMPRSS2 levels do also not reliable predict cell susceptibility to SARS-CoV-2.

### 3.4 Differences between SARS-CoV-2 and SARS-CoV S (Spike) protein cleavage sites and sensitivity to protease inhibitors

R667 and R797 are the critical cleavage sites in SARS-CoV S that are recognized by TMPRSS2 (Simmons *et al.*, 2013; Zhou *et al.*, 2015). These cleavage sites are conserved in SARS-CoV-2 (R685 and R815) (Fig. 1D). However, there is a four amino acid insertion in SARS-CoV-2 S prior to R685 and many of the residues close to R685 are DCPs (V663 = Q677, S664 = T678, T669 = V687, Q671 = S689, K672 = Q690 DCPs are represented by the SARS-CoV residue followed by the SARS-CoV-2 residue) (Fig. 1D). The R815 cleavage site has two DCPs in close proximity (L792 = S810, T795 = S813) (Fig. 1D). Around the R685 cleavage site two DCPs retain polar side chains (S664 = T678, Q671 = S689), while the others represent larger changes between hydrophobic and polar side chains (V663 = Q677, T669 = V687) and one changes from a positive charge to a polar side chain (K672 = Q690). While around the R815 cleavage site, one substitution is conservative (T795 = S813) and the other is a hydrophobic to polar change (L792 = S810).

These changes are likely to impact on TMPRSS2-mediated S cleavage. Indeed, SARS-CoV-2 was more sensitive than SARS-CoV to inhibition by the serine protease inhibitors camostat and nafamostat (Fig. 1E, Supplementary Fig. S6), which are known to inhibit TMPRSS2-mediated S cleavage and virus entry (Hoffmann *et al.*, 2020a,b; Zhou *et al.*, 2015). This confirms that the observed differences in the amino acid sequence of S have functional consequences.

### 3.5 Differences between SARS-CoV-2 and SARS-CoV S interaction with ACE2

Our computational analysis detected further interesting changes in the S protein. SARS-CoV-2 S is 77.46% sequence identical to the SARS-CoV S and many of the remaining positions are DCPs (186 residues) (Supplementary Table S1).

**Fig 2:**
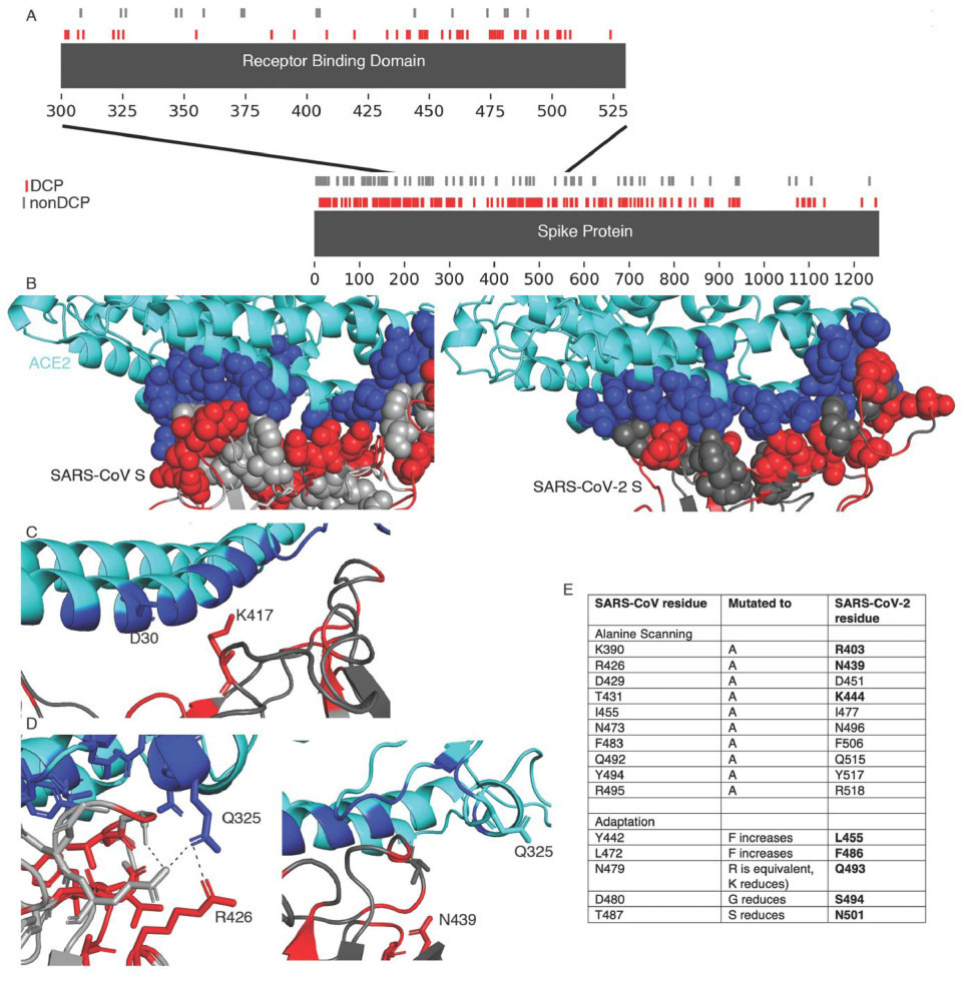
SARS-CoV-2 and SARS-CoV S interaction with ACE2. (**A–D**)Differentially conserved positions in the Spike protein. (A) A sequence view of the DCPs present in the Spike protein, with an inset showing the receptor binding domain. (B) The S interface with ACE2 (cyan). The ACE2 interface is shown in blue spheres, DCPs in red. (C) The V404 = K417 DCP. (D) The R426 = N439 DCP, the left image shows SARS-CoV S R426, the image on the right show the equivalent N439 in SARS-CoV-2 S. (**E**) SARS-CoV residues associated with altering ACE2 affinity and the residues at these positions in SARS-CoV-2 S. (**F**) Cytopathogenic effect (CPE) formation in SARS-CoV-2 and SARS-CoV (MOI 0.01)-infected Caco2 cells in the presence of antibodies directed against ACE2 or DPP4 (MERS-CoV receptor) 48 h post-infection

The SARS-CoV S receptor binding domain (residues 306-527, equivalent to 328-550 in SARS-CoV-2) is enriched in DCPs, containing 43 DCPs (19% of residues). Nine of the 24 SARS-CoV S residues in direct contact with ACE2 were DCPs (Fig. 2A, Supplementary Table S4). Five of these DCPs represent conservative substitutions in amino acid (hydrophobic—hydrophobic or polar-polar), two hydrophobic -polar substitutions, one positive charge to polar change, while the ninth is substitution between a hydrophobic and positively charged amino acid (Supplementary Table S5).

Analysis of the DCPs using the SARS-CoV and SARS-CoV-2 S protein complexes with ACE2 (Song *et al.*, 2018; Yan *et al.*, 2020) identified runs of DCPs (A430-T433, F460-A471) in surface loops forming part of the S-ACE2 interface and resulted in different conformations in SARS-CoV-2 S compared to SARS-CoV S (Figure 2A, 2B). Two DCPs remove intramolecular hydrogen bonding within the spike protein in SARS-CoV-2 (Supplementary Table S4) and three DCPs (R426 = N439, N479 = QQ493, Y484 = Q498) are residues that form hydrogen bonds with ACE2. For two of these positions, hydrogen bonding with ACE2 is present with both S proteins, but for R426 = N439 hydrogen bonding with ACE2 is only observed with SARS-CoV S. N439 in SARS-CoV-2 S is not present in the interface and the sidechain points away from the interface. Further, analysis of the SARS-CoV-2 S-ACE2 complex highlighted important roles of the V404 = K417 DCP, where K417 in SARS-CoV-2 S is able to form a salt bridge with ACE2 D30 (Figure 2C, 2D) (Yan *et al.*, 2020).

Alanine scanning (Chakraborti*et al.*, 2005) and adaptation experiments (Wan *et al.*, 2020) have identified 16 SARS-CoV S residues impacting on the binding affinity with ACE2. For all five residues identified from adaptation studies and four of the 11 identified by alanine scanning experiments, different amino acids are present in SARS-CoV-2 S (Fig. 2E), highlighting the difference in the interaction with ACE2.

In agreement with our structural analysis, we detected differences in the effects of an anti-ACE2 antibody on SARS-CoV-2 and SARS-CoV infection. Antibodies directed against ACE2 were previously shown to inhibit SARS-CoV replication (Li *et al.*, 2003). In line with this, an anti-ACE2 antibody inhibited SARS-CoV infection in Caco2 cells (Fig. 2F). In contrast, the anti-ACE2 antibody displayed limited activity against SARS-CoV-2 infection (Fig. 2F). This shows that it is more difficult to antagonize SARS-CoV-2 infection with anti-ACE2 antibodies and supports previous findings indicating a stronger binding affinity of SARS-CoV-2 S to ACE2 compared to SARS-CoV S (Walls *et al.*, 2020; Wrapp*et al.*, 2020). As anticipated, antibodies directed against DPP4, the MERS-CoV receptor (Cui *et al.*, 2019; de Wit *et al.*, 2016), did not interfere with SARS-CoV or SARS-CoV-2 infection (Fig.2F).

## 4 Discussion

Here, we performed an in-silico analysis of the effects of differentially conserved amino acid positions (DCPs) between SARS-CoV-2 and SARS-CoV proteins on virus protein structure and function in combination with a comparison of wild-type SARS-CoV-2 and SARS-CoV in cell culture.

We identified 891 DCPs, which represents 64% of the amino acid positions that differ between SARS-CoV-2 and SARS-CoV and nearly 9% of all residues encoded by the SARS-CoV genome. 49 of these DCPs are likely to have a structural and functional impact. The DCPs are not equally distributed between the proteins. DCPs are enriched in S, 3a, p6, nsp2, papain-like protease and nsp4, but very few DCPs are present in the envelope (E) protein and most of the remaining non-structural proteins encoded by ORF1ab. This indicates that the individual proteins differ in their tolerance to sequence changes and/or their exposure to selection pressure exerted by the host environment.

The large proportion of DCPs reflects the differences in the clinical behaviour of SARS-CoV-2 and SARS-CoV. Mortality associated with SARS-CoV is higher than that associated with SARS-CoV-2 (Borges do Nascimento, 2020; Cui *et al.*, 2019). SARS-CoV causes a disease of the lower respiratory tract. Infected individuals are only contagious when they experience symptoms (de Wit *et al.*, 2016). SARS-CoV-2 is present in the upper respiratory tract and can be readily transmitted prior to the onset of symptoms. Mild but infectious cases may substantially contribute to its spread (Rivett*et al.*, 2020).

The large proportion of DCPs reflects the differences in the clinical behaviour of SARS-CoV-2 and SARS-CoV. Mortality associated with SARS-CoV is higher than that associated with SARS-CoV-2 (Borges do Nascimento, 2020; Cui *et al.*, 2019). SARS-CoV causes a disease of the lower respiratory tract. Infected individuals are only contagious when they experience symptoms (de Wit *et al.*, 2016). SARS-CoV-2 is present in the upper respiratory tract and can be readily transmitted prior to the onset of symptoms. Mild but infectious cases may substantially contribute to its spread (Rivett*et al.*, 2020).

Although further research will be required to elucidate in detail, which DCPs are responsible for which differences in virus behaviour, our analysis has already provided important clues. Both viruses use ACE2 as a receptor and are activated by the transmembrane serine protease TMPRSS2 (Cui *et al.*, 2019; Hoffmann *et al.*, 2020a; Li *et al.*, 2003; Walls*et al.*, 2020; Wan *et al.*, 2020; Wrapp*et al.*, 2020; Yan *et al.*, 2020). Our results show, however, that the ACE2 and the TMPRSS2 status are not sufficient to predict cells susceptibility to SARS-CoV-2 or SARS-CoV. The cell line CL14 supported SARS-CoV-2 replication, although it displayed lower ACE2 levels and similar TMPRSS2 levels to non-susceptible DLD-1 and HT29 cells. Thus, attempts to identify SARS-CoV-2 target cells based on the ACE2 status (Luan *et al.*, 2020; Qiu*et al.*, 2020; Xuet al., 2020) need to be considered with caution.

As previously described (Kamitani*et al.*, 2006), ACE2 expression rendered SARS-CoV non-permissive 293 cells susceptible to SARS-CoV. However, ACE2 expression had a substantially lower impact on SARS-CoV-2 infection. This suggests the presence of further host cell factors that determine SARS-CoV-2 susceptibility. Based on our sequence analysis, DCPs in the viral interferon antagonists may contribute to the differences observed in the cellular tropism of SARS-CoV-2 and SARS-CoV.

Our computational analysis detected DCPs in the ACE2-binding domain of S, which are likely to impact S-ACE2 binding. In agreement, an anti-ACE2 antibody displayed higher efficacy against SARS-CoV than against SARS-CoV-2, illustrating the differences between SARS-CoV-2 S and SARS-CoV S interaction with ACE2. This probably reflects an increased SARS-CoV-2 S affinity to ACE2 compared to SARS-CoV S (Wrapp*et al.*, 2020), which may be more difficult to antagonize.

To mediate virus entry, S needs to be cleaved by host cell proteases, in particular by TMPRSS2 (Hoffmann *et al.*, 2020a,b; Zhou *et al.*, 2015). The S cleavage sites are conserved between SARS-CoV-2 and SARS-CoV. However, we found DCPs in close vicinity to the S cleavage sites, which are likely to affect S cleavage by host cell enzymes and/or the activity of protease inhibitors on S cleavage. Indeed, the serine protease inhibitors camostat and nafamostat, which interfere with S cleavage (Hoffmann *et al.*, 2020a,b), displayed increased activity against SARS-CoV-2 infection than against SARS-CoV infection, confirming the functional relevance of the DCPs.

In conclusion, our in-silico study revealed a substantial number of differentially conserved amino acid positions in the SARS-CoV-2 and SARS-CoV proteins. In agreement, cell culture experiments indicated differences in the cell tropism of these two viruses and showed that cellular ACE2 and TMPRSS2 levels do not reliably indicate cell susceptibility to SARS-CoV-2. Moreover, we identified DCPs in S that are associated with differences in the interaction with ACE2 and increased SARS-CoV-2 sensitivity to the protease inhibitors camostat and nafamostat relative to SARS-CoV.

## Supplementary Material

btab094_Supplementary_Datay
