# Supplementary material for: Differentially conserved amino acid positions may reflect differences in SARS-CoV-2 and SARS-CoV behaviour
Source: Bioinformatics. 2021 Feb 9;37(16):2282–8. doi: 10.1093/bioinformatics/btab094 (PMC7929367; doi:10.1093/bioinformatics/btab094)
Supplement: btab094_Supplementary_Datay [file btab094_supplementary_datay.zip › Bojkova et al_Supplements_002.pdf]

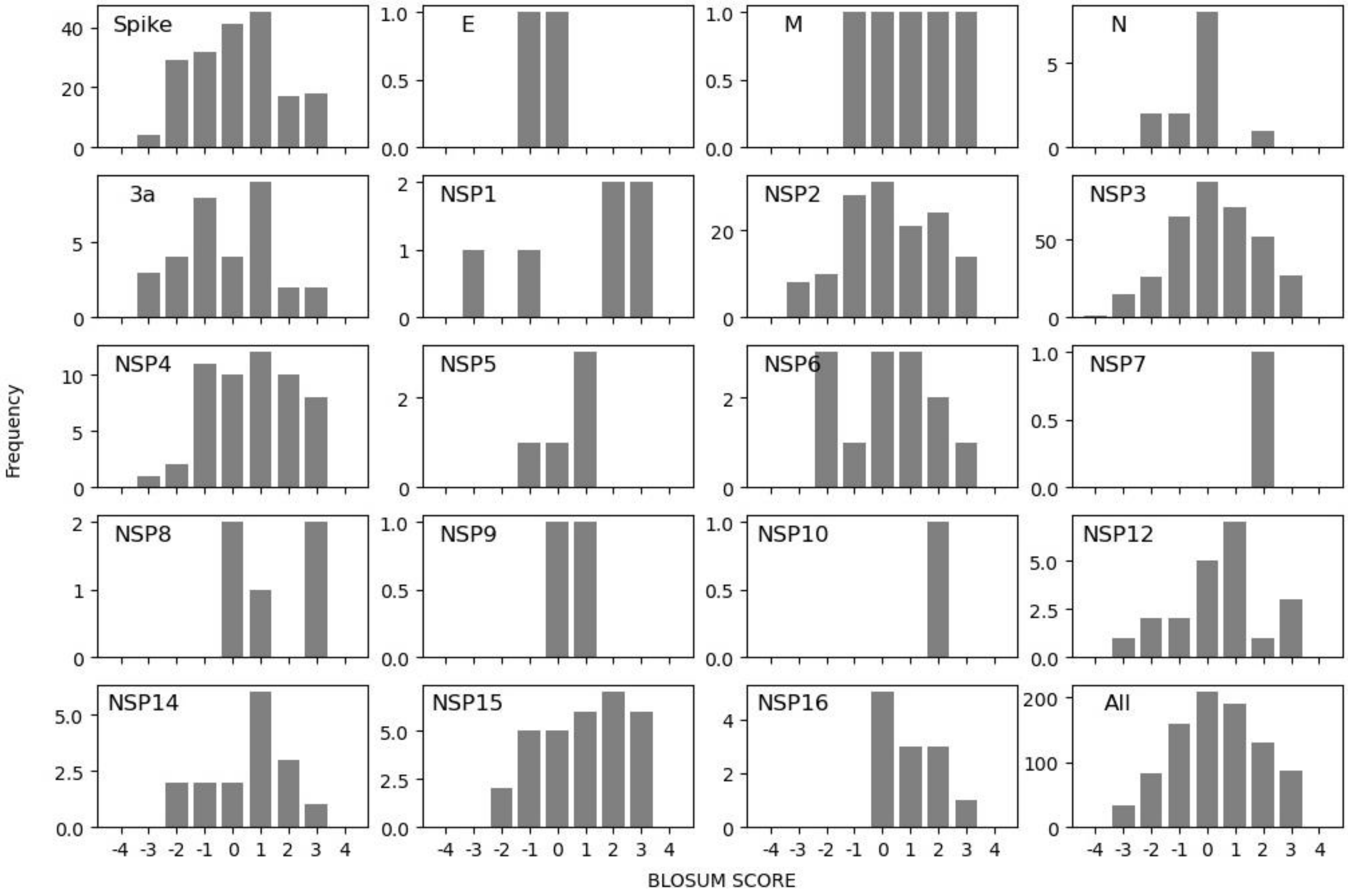

Figure S1. The BLOSUM scores for the amino acid substitutions present in the SDPs. A graph is plotted that combines all of the proteins and one for each of the individual proteins that were analysed.

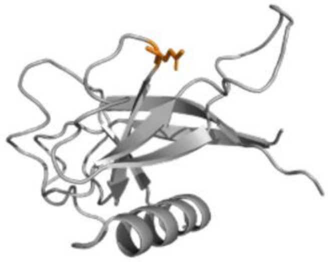

NSP1 (Phyre 2 model d2gdta1

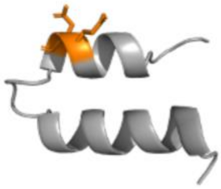

NSP1

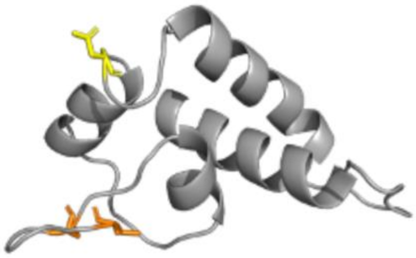

NSP4 (Phyre2 model c4gzfD)

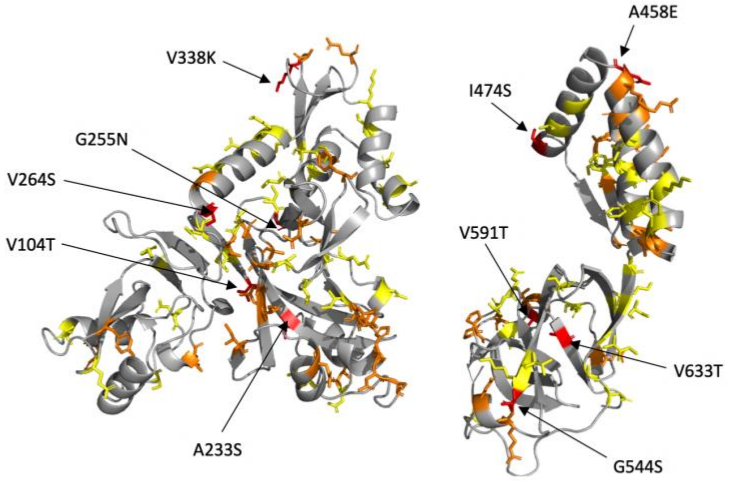

NSP2 (AlphaFold model)

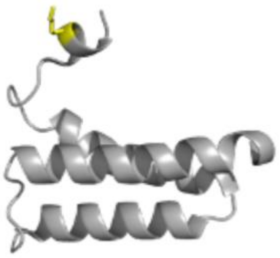

NSP7 (6xip)

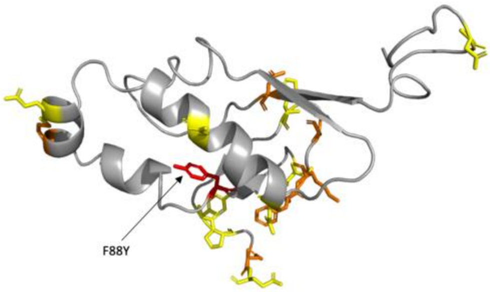

NSP3 (Phyre2 d2grla1)

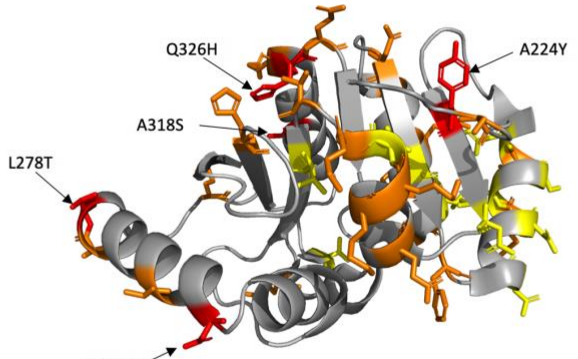

NSP3 (6vxs)

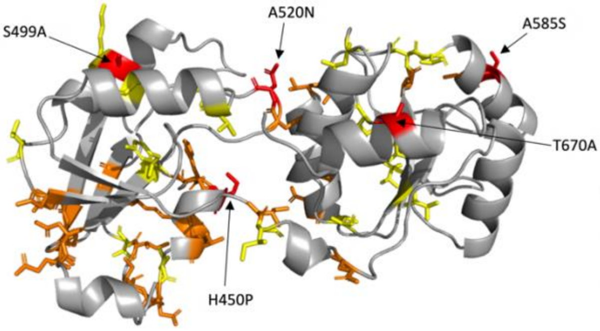

NSP3 (Phyre2 c2wctC)

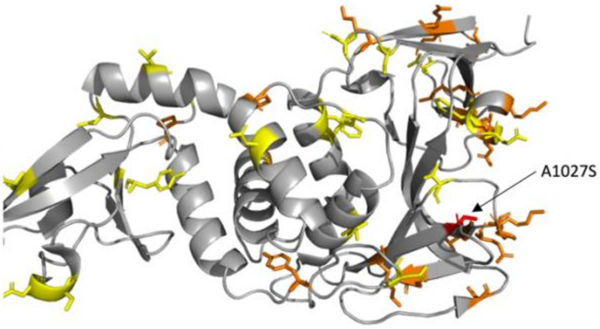

NSP3 (6w9c)

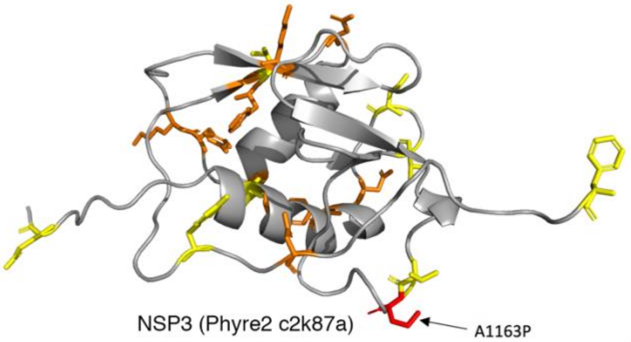

NSP3 (Phyre2 c2k87a)

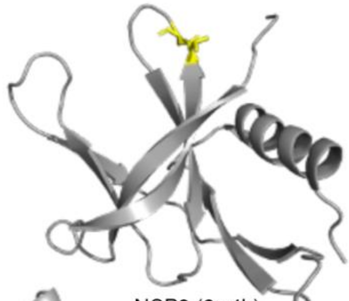
NSP9 (6w4b)

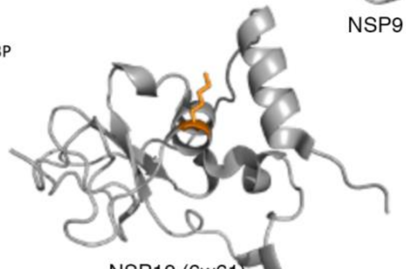
NSP10 (6w61)

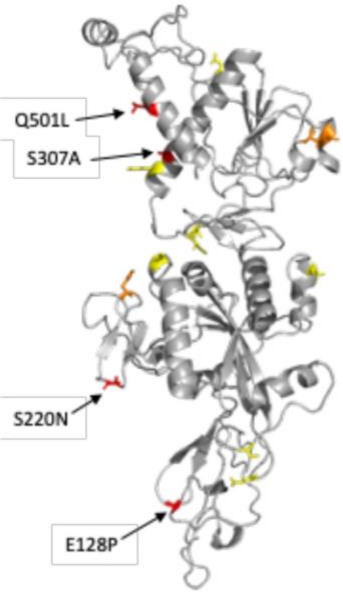

NSP14 (Phyre2 c5c8sd)

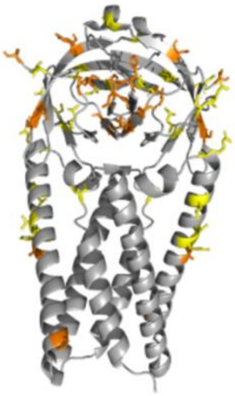
3a (6xdc)

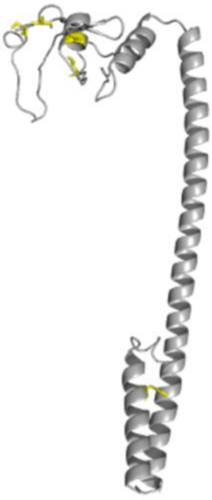
NSP8 (Phyre2 c2ahmG)

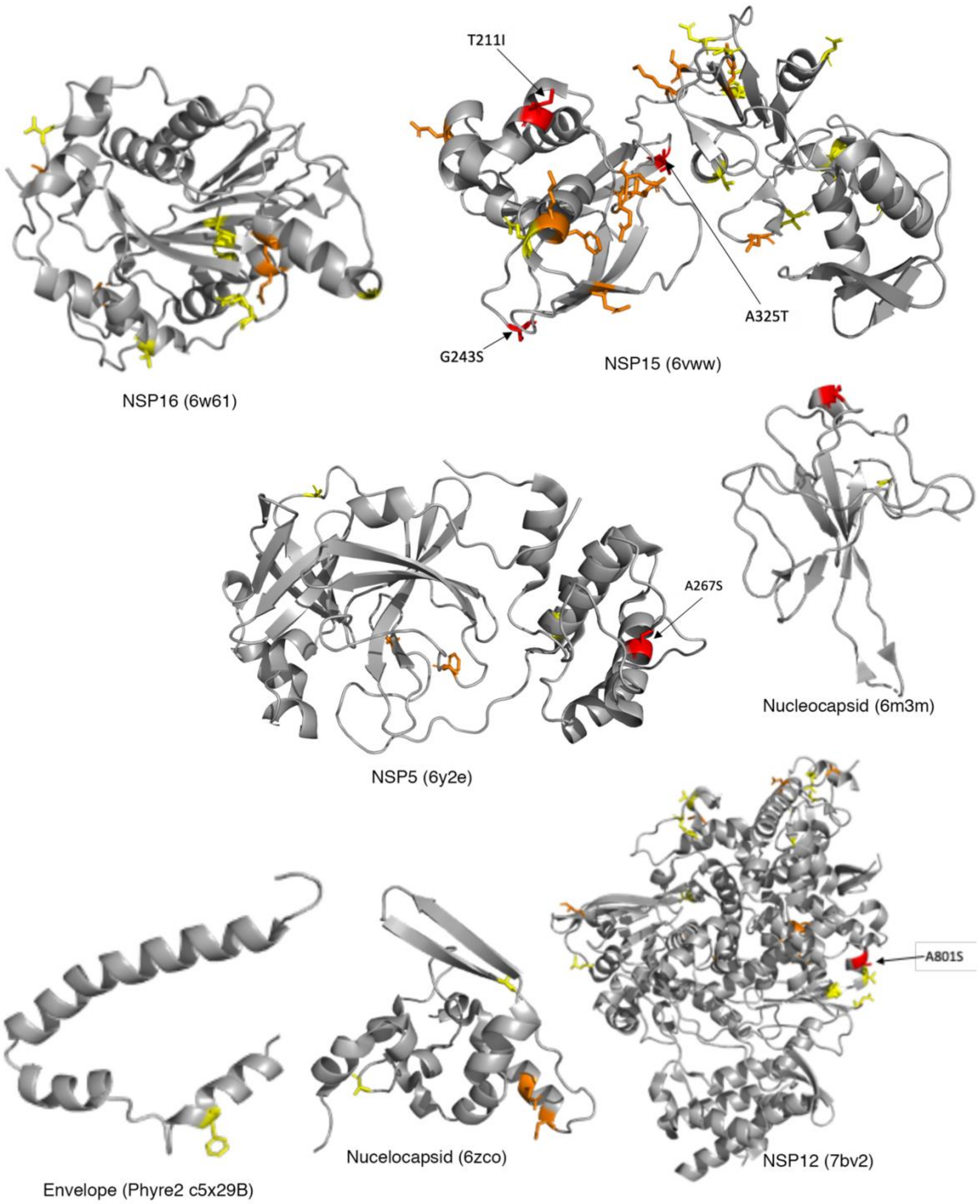
T211I
A325T
G243S
NSP16 (6w61)
NSP15 (6vww)
A267S
Nucleocapsid (6m3m)
NSP5 (6y2e)
A801S
Envelope (Phyre2 c5x29B)
Nucelocapsid (6zco)
NSP12 (7bv2)

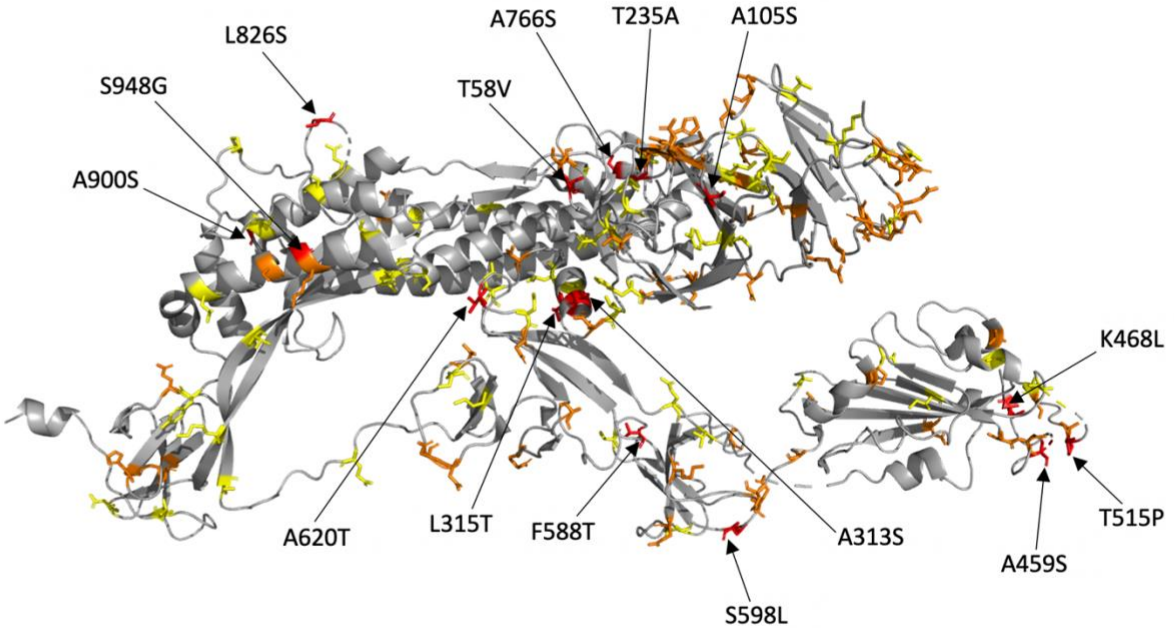

Figure S2. Overview of modelled DCPs. DCPs with likely functional effect are indicated by arrows and labelled. Structural model shown is indicated in brackets. DCPs likely to have an effect are coloured red; DCPs with a possible effect are shown in orange; and DCPs unlikely to have an effect are coloured yellow. Please refer to table S6 for full details of structural analysis of each DCP.

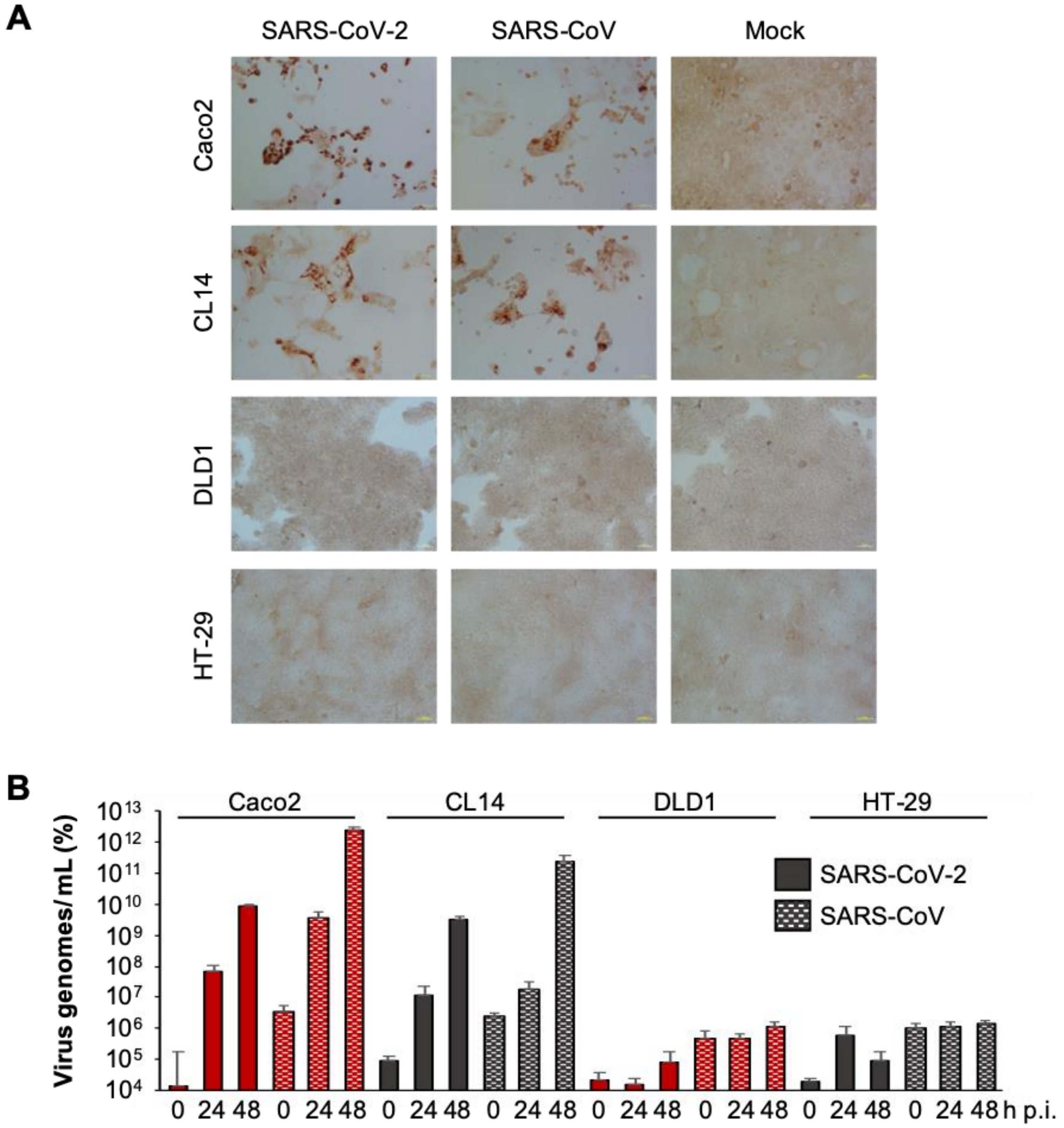

Figure S3. SARS-CoV-2 and SARS-CoV susceptibility of cell lines. A) Representative images showing MOI 0.01-infected cells immunostained for double-stranded RNA 48h post infection. B) Quantification of virus genomes by qPCR at different time points post infection (p.i.). Values are presented as means ± S.D. (n =3).

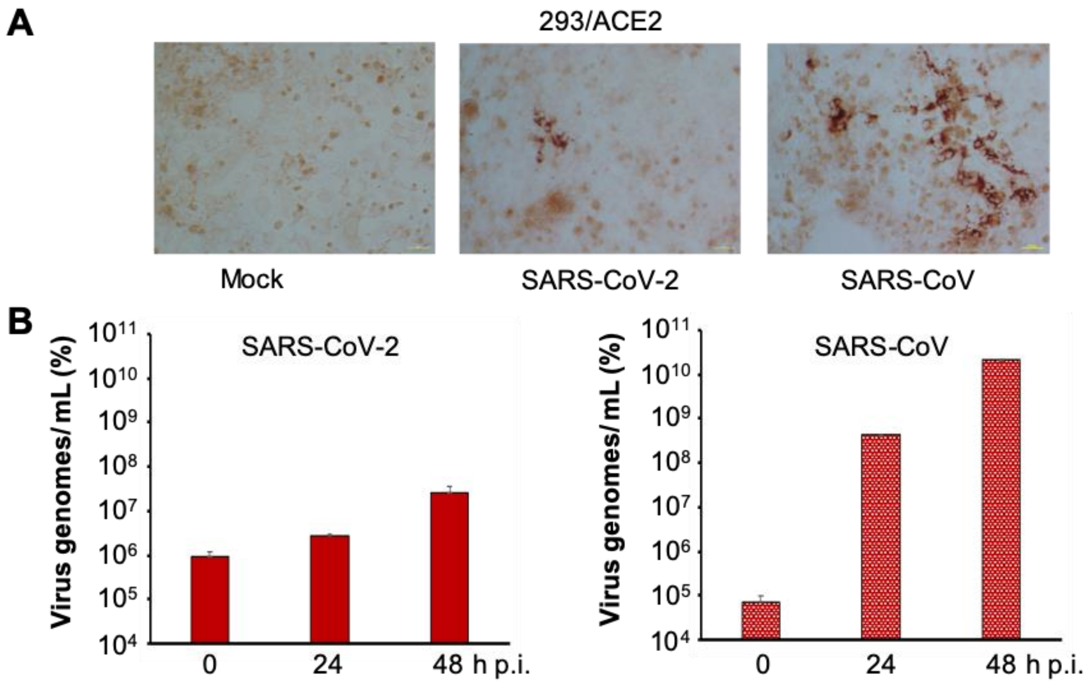

Figure S4. SARS-CoV-2 and SARS-CoV replication in 293 cells stably expressing ACE2 cells (293/ACE2). A) Immunostaining for double-stranded RNA (indicating virus replication) in SARS-CoV-2 and SARS-CoV (MOI 0.01)-infected 293/ACE2 cells 48h post infection. B) Quantification of virus genomes by qPCR in SARS-CoV-2 and SARS-CoV (MOI 0.01)-infected 293/ACE2 cells 48h post infection. Values are presented as means ± S.D. (n =3).

Figure 1C

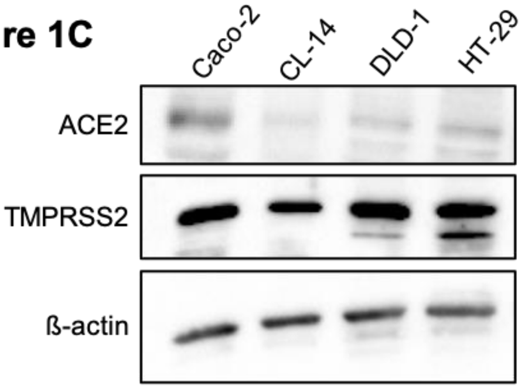

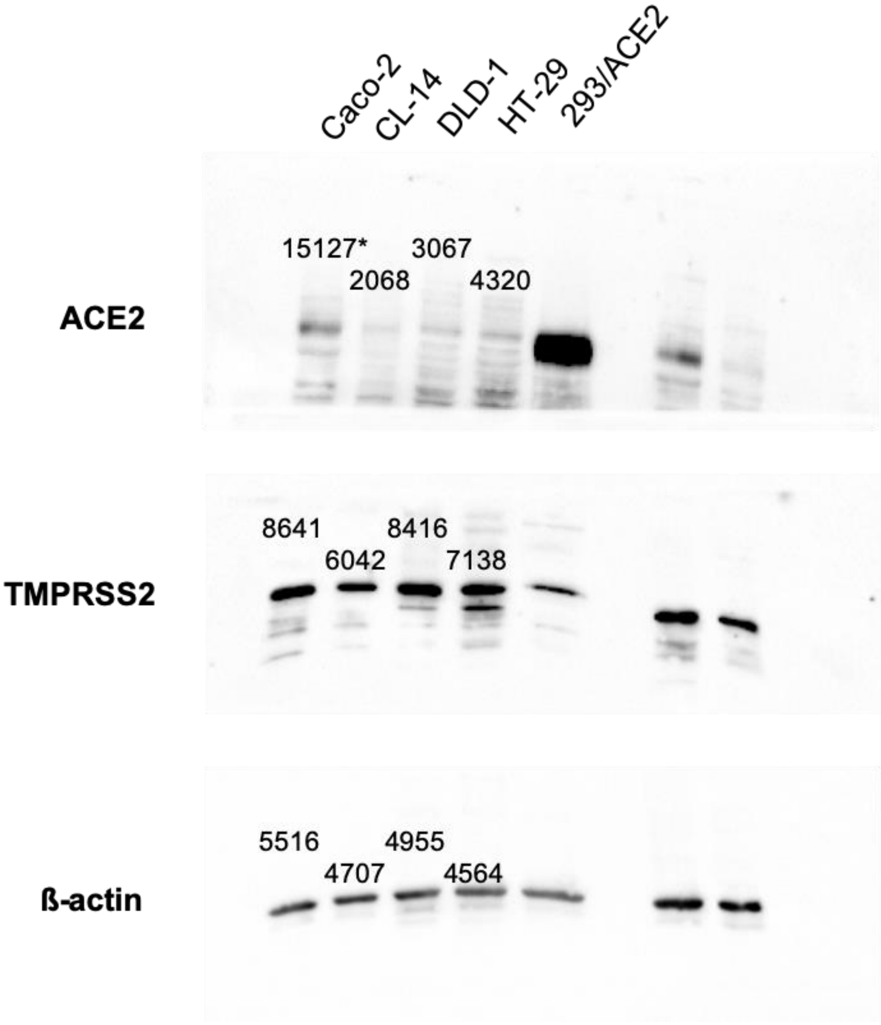

Figure S5. Uncropped Western blots for Figure 2D. 293/ACE2 cells served as positive control for ACE2. * Protein quantification

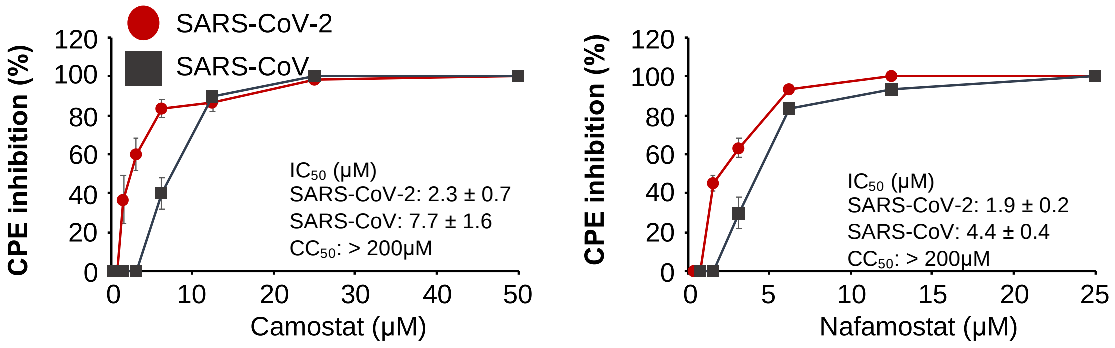

Figure S6. Role of TMPRSS2-mediated S cleavage in SARS-CoV-2 and SARS-Co-V replication. Concentration-dependent effects of the TMPRSS2 inhibitors camostat and nafamostat on SARS-CoV-2- and SARS-CoV-induced cytopathogenic effect (CPE) formation determined 48h post infection in CL14 cells infected at an MOI of 0.01. Values are presented as means ± S.D. (n =3).

Table S1. Protein structures used for structural analysis obtained from the Protein Databank.

SARS	PDB identifier	Protein	Residues
SARS-CoV	2hsx	NSP1	13-127
SARS-CoV	2gri	NSP3	1-111
SARS-CoV	2fav	NSP3	185-354
SARS-CoV-2	6vxs	NSP3	207-373
SARS-CoV	2w2g	NSP3	389-652
SARS-CoV	2kaf	NSP3	655-720
SARS-CoV	5y3e	NSP3	723-1036
SARS-CoV-2	6w9c	NSP3	748-1061
SARS-CoV	2k87	NSP3	1066-1180
SARS-CoV	2h2z	NSP5	1-306
SARS-CoV-2	6y2e	NSP5	1-306
SARS-CoV	6nur	NSP7	2-71
SARS-CoV-2	6xip	NSP7	1-70
SARS-CoV	2ahm	NSP8	1-190
SARS-CoV	2fyg	NSP10	10-132
SARS-CoV-2	6w61	NSP10	18-132
SARS-CoV	6nur	NSP12	41-819
SARS-CoV-2	7bv2	NSP12	31-929
SARS-CoV	5c8s	NSP14	1-525
SARS-CoV	2h85	NSP15	1-345
SARS-CoV-2	6vww	NSP15	1-346
SARS-CoV	2xyq	NSP16	1-290
SARS-CoV-2	6w61	NSP16	1-299
SARS-CoV	6acg	S:ACE2	18-1119
SARS-CoV-2	6m17	S:ACE2	336-518
SARS-CoV	5xlr	S	33-1120
SARS-CoV	5wrg	S	261-1058
SARS-CoV-2	6vsb	S	27-1146
SARS-CoV-2	6xdc	3a	40-238
SARS-CoV	5x29	E	8-65
SARS-CoV	1yo4	7a	16-99
SARS-CoV	1ssk	N	49-185
SARS-CoV-2	6m3m	N	48-173
SARS-CoV	2gib	N	270-366
SARS-CoV-2	6zco	N	248-364

Table S2. Structural models generated by Phyre2 and used for structural analysis. Where structures were not available from the Protein Databank, the structures were modelled.

SARS	Template structure	Protein	Residues	Coverage	Confidence	Identity (%)
SARS-CoV-2	2gdta1	NSP1	13-127	64	100	86
SARS-CoV	6zojj	NSP1	148-180	17	99.1	76
SARS-CoV-2	6zojj	NSP1	148-180	18	99.8	100
SARS-CoV-2	2gria1	NSP3	2-111	5	100	77
SARS-CoV-2	2acfa1	NSP3	207-373	8	100	74
SARS-CoV-2	2wctC	NSP3	425-676	12	100	76
SARS-CoV-2	2fe8B	NSP3	745-1058	16	100	82
SARS-CoV-2	2k87A	NSP3	1089-1203	5	100	82
SARS-CoV-2	3gzfD	NSP4	403-477	18	100	41
SARS-CoV-2	2duca1	NSP5	2-283	98	100	96
SARS-CoV-2	2ahmG	NSP8	1-175	95	100	97
SARS-CoV-2	1uw7A	NSP9	1-90	100	100	97
SARS-CoV-2	2g9tT	NSP10	9-116	86	100	98
SARS-CoV-2	6nusA	NSP12	118-909	87	100	97
SARS-CoV-2	5c8sD	NSP14	1-504	97	100	95
SARS-CoV	6xdcB	3a	40-238	72	100	77
SARS-CoV-2	5x29B	E	8-65	77	99.8	91
SARS-CoV-2	1yo4A	7a	16-98	67	100	91

Table S3. Criteria used for classifying proposed effect on protein structure and function within the structural analysis.

Effect	Reason
Unlikely	Conservative changes (between residues with the same polarity/charge) which do not affect ability to form hydrogen bonds with equivalent residues in SARS-CoV and SARS-CoV-2
Possible – conformational change	Changes which could affect the ability of a sidechain of a residue in a given position to form hydrogen bonds with equivalent residues in SARS-CoV and SARS-CoV-2 (e.g. gain/loss of polarity, substitution for larger/smaller sidechain) but no such effects are visible, or conservative changes (between residues with the same polarity/charge) which appear in the model to result in gain/loss of hydrogen bonding between equivalent residues in SARS-CoV and SARS-CoV-2 (but mutagenesis suggests hydrogen bonding is possible with sidechain rotation)
Possible – alteration of sidechain/ligand interactions	Changes which result in gain of charge/alter the charge of a sidechain for a residue in a given position
Possible – conformational change and alteration of sidechain/ligand interactions	Changes which affect the ability of a sidechain of a residue in a given position to form hydrogen bonds with equivalent residues in SARS-CoV and SARS-CoV-2 (e.g. gain/loss of polarity, substitution for larger/smaller sidechain) but no such effects are visible, and changes which result in gain of charge/alter the charge of a sidechain for a residue in a given position
Likely – conformational change	Changes which result in visible alteration in the conformation of a protein at a given location (e.g. through loss of hydrogen bonding between equivalent residues in SARS-CoV and SARS-CoV-2) and/or which result in the loss of capacity for hydrogen bonding
Likely – conformational change (and possible alteration of sidechain/ligand interactions)	Changes which result in visible alteration in the conformation of a protein at a given location (e.g. through loss of hydrogen bonding between equivalent residues in SARS-CoV and SARS-CoV-2, and/or which result in the loss of capacity for hydrogen bonding and which result in gain of charge/alter the charge of a sidechain for a residue in a given position)

Table S4. Specificity Determining Positions (DCPs) identified between SARS-CoV and SARS-CoV-2.

**Protein (SARS-CoV)**	**Protein (SARS-CoV-2)**	**Sequences in Dataset**	**Protein Length (SARS-CoV)**	**DCPs Identified**	**% of Residues DCPs**
S	S	73863	1255	186	14.82
3a	ORF3a	91214	274	32	11.68
3b		n/a	154		
E	E	94787	76	2	2.63
M	M	93860	221	15	2.26
6	6	94935	63	13	9.52
7a	7a	82940	122	0	0
7b	7b	n/a	44	NA	
8a/8b	8	n/a	39/84	NA	NA
9b		n/a	98	NA	
N	N	91609	422	13	3.08
	ORF10	n/a	n/a		
nsp1	nsp1	93621	180	6	3.33
nsp2	nsp2	88288	636	136	21.38
Nsp3	nsp3	75324	1922	344	17.90
nsp4	nsp4	89707	500	54	10.80
nsp5	nsp5	91731	306	5	1.63
nsp6	nsp6	93432	290	13	4.48
nsp7	nsp7	95038	83	1	1.20
nsp8	nsp8	94806	198	5	2.53
nsp9	nsp9	94970	113	2	1.77
nsp10	nsp10	92505	139	1	0.72
nsp12	nsp12	89874	932	21	2.25
nsp13	nsp13	91305	601	0	0
nsp14	nsp14	72306	527	16	3.04
nsp15	nsp15	85595	346	31	8.96
nsp16	nsp16	83565	298	12	4.03
Total				891	9.36

Table S5 - Analysis of DCPs present in the SARS-CoV and SARS-CoV-2 Spike protein interface with human ACE2.

SDP	SARS-CoV structural analysis	SARS-CoV-2 structural analysis	Effect?
V404=K417	V404 is not in the interface	K417 is in the interface and could form a salt bridge with ACE-2 D30	Likely – new polar interaction within interface
R426=N439	Loss of hydrogen bond to ACE2 Gln325 due to shorter sidechain. N would still be able to form hydrogen bonds	N439 is located away from the interface site and so does not form a hydrogen bond with ACE2. Instead forms a hydrogen bond with S443 (also a DCP – A430=S443) which is likely to stabilise the loop they are both part of.	Likely – Loss of interface hydrogen bond.
Y442=L455	Y422 forms hydrogen bond to backbone of W476 – loss could result in conformational change. The sidechain also contacts the backbone of ACE2 D30 and K31	L455 remains in interface and contacts ACE2 D30 and H34.	Likely – loss of intramolecular hydrogen bond
F460=Y473	Conservative change.	Introduction of OH group that can form hydrogen bonds. Y473 forms hydrogen bond with backbone of R457 and is closer to ACE2 T27 so potential to form hydrogen bond in interface.	Possible – introduction of hydrogen bond (could be with ACE2)
P462=A475	Located in a loop, could affect this conformation – many DCPs in this loop	Loop has different conformation.	Possible – Conformational change of loop
N479=Q493	Interface hydrogen bond formed with ACE2 H34 backbone. With a shorter sidechain this this may be lost in SARS-CoV-2.	Q493 forms a hydrogen bond with ACE2 E35 in this complex. So hydrogen bond is maintained but also different.	Possible – hydrogen bond with ACE2 retained but to different residue.
Y484=Q498	Y484 can form hydrogen bonds with ACE2 Gln42 (sidechain) and intramolecular H bonds with T433 (backbone), Y436 (sidechain).	Q498 maintains hydrogen bonds with ACE2 Gln42	Possible – change in residue forming hydrogen bonds with ACE2.

T485=P499	Sidechain points away from interface, loss of hydrogen bond with R426 (also a DCP) backbone in adjacent loop. This hydrogen bond is likely to coordinate the structure between these two loops. There are multiple DCPs present in both loops	Loop conformation similar as for SARS-CoV structure but not coordinated with other loop	Likely - loss of intramolecular hydrogen bond
I489=V503	Conservative change I489 in direct contact with ACE2 Q325	slightly smaller sidechain is further away from ACE2 Q325.	Unlikely.
